# Staged bilateral reverse sural artery flap reconstruction for severe frostbite of both feet in a patient with schizophrenia: A case report

**DOI:** 10.1097/MD.0000000000051122

**Published:** 2026-10-09

**Authors:** Jae Hyun Kim, Myeong Seop Lee, Chan Min Chung

**Affiliations:** a Department of Plastic and Reconstructive Surgery, Hallym Sacred Heart Hospital, Hallym University College of Medicine, Anyang, Korea.

**Keywords:** Frostbite, Limb Salvage, Schizophrenia, Surgical Flaps, Reverse sural artery flap

## Abstract

**Rationale:**

Bilateral, weight-bearing heel defects after frostbite are difficult to reconstruct, and outcomes depend heavily on postoperative off-loading and patient cooperation. We report a limb-salvage strategy for severe bilateral foot frostbite that occurred during acute psychosis in a patient diagnosed with schizophrenia.

**Patient concerns:**

A 25-year-old man developed acute psychotic symptoms and walked barefoot in subzero conditions for about 12 hours, resulting in deep frostbite of both heels and multiple toes. During admission he had poor insight and repeatedly attempted weight bearing despite instructions.

**Diagnoses:**

MRI/CT showed necrosis of the right first to third toes and bilateral posterior heel soft-tissue necrosis with preserved deep heel structures, supporting flap-based salvage rather than major amputation. Schizophrenia was diagnosed and treated concurrently.

**Interventions:**

Reconstruction was staged. The right first to third toes were amputated, the right heel was debrided, and coverage was achieved with a reverse sural artery flap (RSAF). Three weeks later, the left great toe underwent debridement with split-thickness skin grafting, and the left heel defect was reconstructed with an RSAF. Strict positioning/off-loading and close multidisciplinary inpatient supervision were used to protect both flaps while psychiatric symptoms were stabilized.

**Outcomes:**

At the last documented follow-up, 2 weeks after discharge – corresponding to 5 weeks after the left-sided reconstruction and approximately 8 weeks after the right-sided reconstruction – both flaps remained viable, with no total flap loss or flap-related reoperation, and small residual superficial wounds remained. No subsequent clinical follow-up was available.

**Lessons:**

In frostbite patients with severe mental illness, reconstructive choice should balance technical “ideal” options against real-world feasibility. In this patient, staged regional flap reconstruction provided short-term wound coverage while accommodating psychiatric stabilization and limited adherence; however, long-term durability could not be assessed.

## 1. Introduction

Frostbite is a localized cold-induced injury that ranges from superficial tissue damage to deep necrosis involving bone and joint structures. The distal extremities are particularly vulnerable because of their exposure and relatively poor insulation, and deep frostbite of the feet is frequently complicated by partial foot amputation. Frostbite is strongly associated with psychosocial and behavioral risk factors. A systematic review has identified alcohol misuse, homelessness, and mental disorder as major predictors of frostbite.^[1]^ Cognitive impairment, poor insight, and disorganized behavior in schizophrenia can lead to prolonged cold exposure, delayed presentation, and later difficulty adhering to complex treatment regimens, all of which increase the risk of deep, limb-threatening injury.

Restoration of soft tissue over the heel is technically demanding because this region must provide stable, sensate, and durable coverage under high mechanical load. Various reconstructive algorithms have been proposed, incorporating local, regional, and free flaps to address defects of differing size, depth, and etiology. Microvascular free flaps, such as the anterolateral thigh or medial plantar perforator flap, are often considered for large or bilateral plantar defects; however, they require long operations, sophisticated postoperative monitoring, and a high degree of patient cooperation. The reverse sural artery flap (RSAF) has its origins in the distally based fasciocutaneous flap from the sural region described by Donski and Fogdestam in 1983.^[2]^ It is an established regional reconstructive option for distal leg, ankle, and heel defects that does not require microvascular anastomosis.

Many patients with schizophrenia require long-term treatment with antipsychotic medications, and perioperative planning should account for potential medication-associated systemic risks. In a meta-analysis, exposure to antipsychotics was associated with an increased risk of venous thromboembolism (VTE), supporting the concept that these agents may contribute to a prothrombotic milieu in susceptible patients.^[3]^ In reconstructive surgery, thrombotic events are a leading mechanism of free-flap compromise; therefore, when microsurgical reconstruction is being considered, any clinical factor that may increase thrombotic tendency – including antipsychotic exposure – can reasonably be incorporated into risk stratification and may influence the selection and staging of the reconstructive strategy, particularly in complex lower-extremity defects where flap failure would have major functional consequences.

Against this background, flap reconstruction for severe frostbite of both feet in a patient with schizophrenia represents an uncommon and particularly challenging clinical scenario. To our knowledge, existing frostbite literature mentions psychiatric illness primarily as a risk factor and provides little guidance on flap-based limb salvage when acute psychiatric instability coexists with bilateral weight-bearing heel defects. This combination is particularly challenging because both reconstructed heels require protection and off-loading, while successful postoperative care depends on sustained patient cooperation. We report a case of bilateral frostbite of the feet in a young man with schizophrenia, in whom we elected staged reconstruction with bilateral RSAFs rather than free tissue transfer. The contribution of this report lies in describing the reconstructive decision-making and postoperative management of bilateral heel defects during acute psychosis, with particular emphasis on staged reconstruction and flap protection in a patient with limited capacity to adhere to postoperative instructions.

## 2. Case presentation

A 25-year-old man with no previous psychiatric or medical history was brought to our institution after sustaining severe cold injury to both feet. Approximately 1 month before admission, he had developed acute psychotic symptoms characterized by prominent auditory hallucinations and fixed delusional ideas, including the belief that he was a “returned messiah.” Under the influence of these psychotic experiences, he traveled alone to Mongolia and, in response to a perceived command to “walk barefoot in the snow,” reportedly walked without shoes for approximately 12 hours in subzero conditions. He eventually collapsed outdoors and was found by local residents, after which he was repatriated to Korea through the consular office. Despite visiting several hospitals after returning, definitive surgical treatment was repeatedly delayed because simultaneous management of his acute psychosis and complex foot wounds was considered difficult.

Eventually, he was admitted to the neuropsychiatric ward of our hospital with a diagnosis of schizophrenia. During hospitalization, his regular psychiatric regimen included olanzapine, quetiapine, and paliperidone, together with clonazepam, benztropine, and divalproex. Initial laboratory tests, including complete blood count, inflammatory markers, and serum chemistry, were within normal reference ranges, with no evidence of systemic infection or organ dysfunction. He was intermittently responding to internal stimuli, engaging in persistent self-talk, and repeatedly insisting on discharge. He exhibited poor insight into both his psychiatric condition and the severity of his foot injuries. Despite instructions to remain non–weight-bearing, he frequently attempted to stand and walk on both feet, placing the injured areas at high risk of further tissue damage. During the early phase of admission, when psychotic symptoms were severe, lorazepam and haloperidol were administered as needed, and wrist restraints were used because he attempted to leave the bed and stand on the injured feet. His behavior gradually improved with psychiatric treatment, and the restraints were discontinued before surgery. No further as-needed lorazepam or haloperidol was administered from several days before the first operation onward; regular psychiatric medications were continued throughout the surgical treatment period. Examination of the affected extremities revealed deep frostbite injuries involving both heels, the left great toe, and the right first, second, and third toes (Fig. 1).

**Figure 1.**
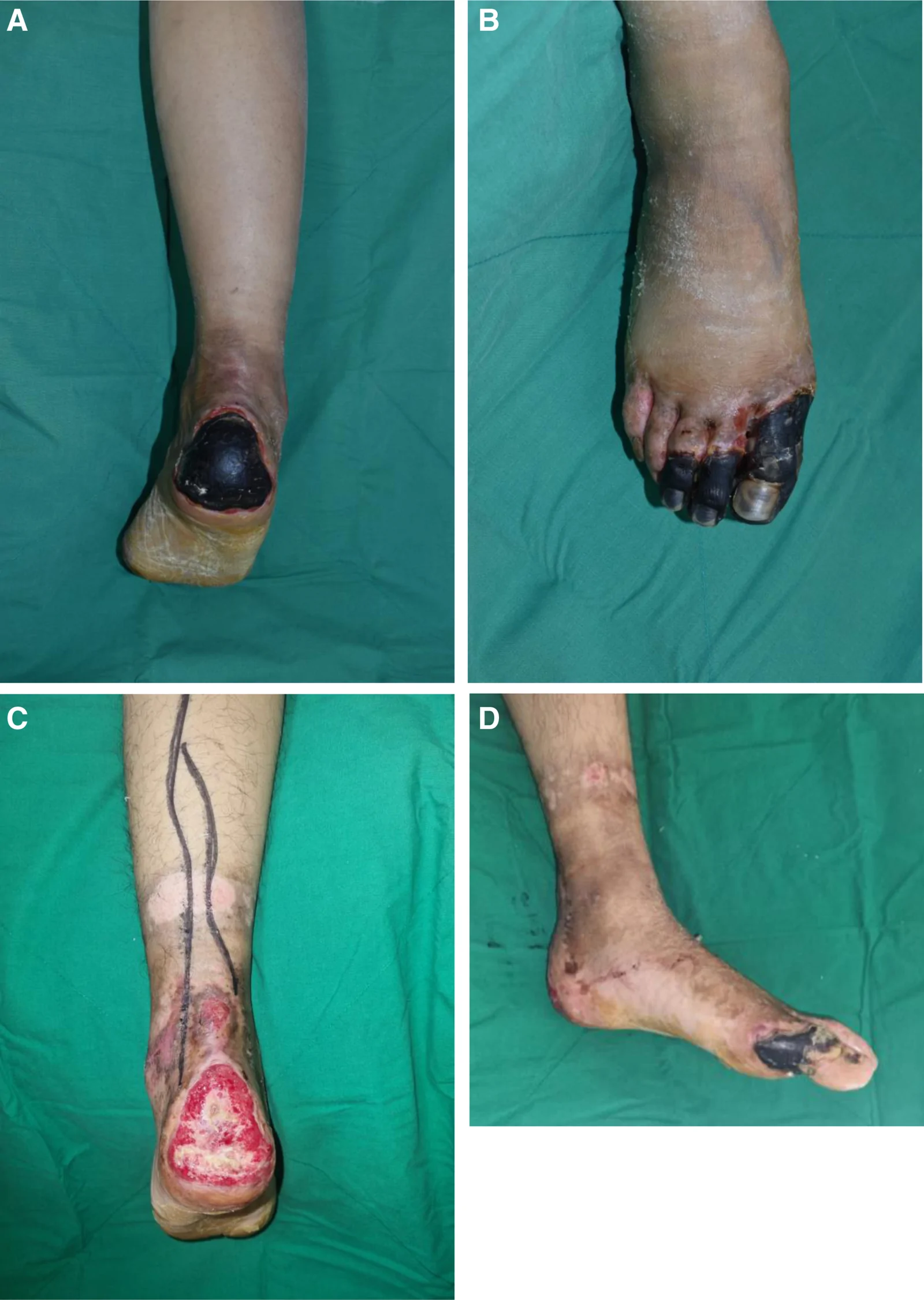
Preoperative findings in a patient with severe bilateral foot frostbite. (A) Posterior view of the right foot demonstrating a large, well-demarcated, dry black eschar involving almost the entire weight-bearing heel. (B) Dorsal view of the right foot showing dry gangrenous changes of the first to third toes with surrounding soft-tissue swelling and skin desquamation. (C) Posterior view of the left heel revealing a large posterior heel defect with healthy granulation tissue. (D) Lateral view of the left foot demonstrating dry necrosis of the first toe.

Given the extent and depth of the injuries, detailed imaging was obtained to accurately assess the damage and to plan appropriate surgical intervention. Contrast-enhanced magnetic resonance imaging (MRI) of both feet, combined with computed tomography (CT), demonstrated bone and soft-tissue necrosis of the right first to third toes. Necrosis involved the proximal and middle phalanges of the great toe and the middle and distal phalanges of the second and third toes, showing lack of contrast enhancement on MRI and intraosseous gas on CT. Cold-injury-related bone marrow inflammation was noted in the calcaneal tuberosity, first, fourth, and fifth distal metatarsals, second to fifth proximal phalanges, and fourth and fifth middle and distal phalanges of the right foot, as well as subcortical bone marrow of the medial and lateral malleoli. On the right heel, focal skin necrosis with surrounding soft-tissue edema was observed, but there was no radiologic evidence of involvement of the calcaneus, plantar fascia, or Achilles tendon. An insufficiency fracture of the posterior calcaneus was also noted (Fig. 2).

**Figure 2.**
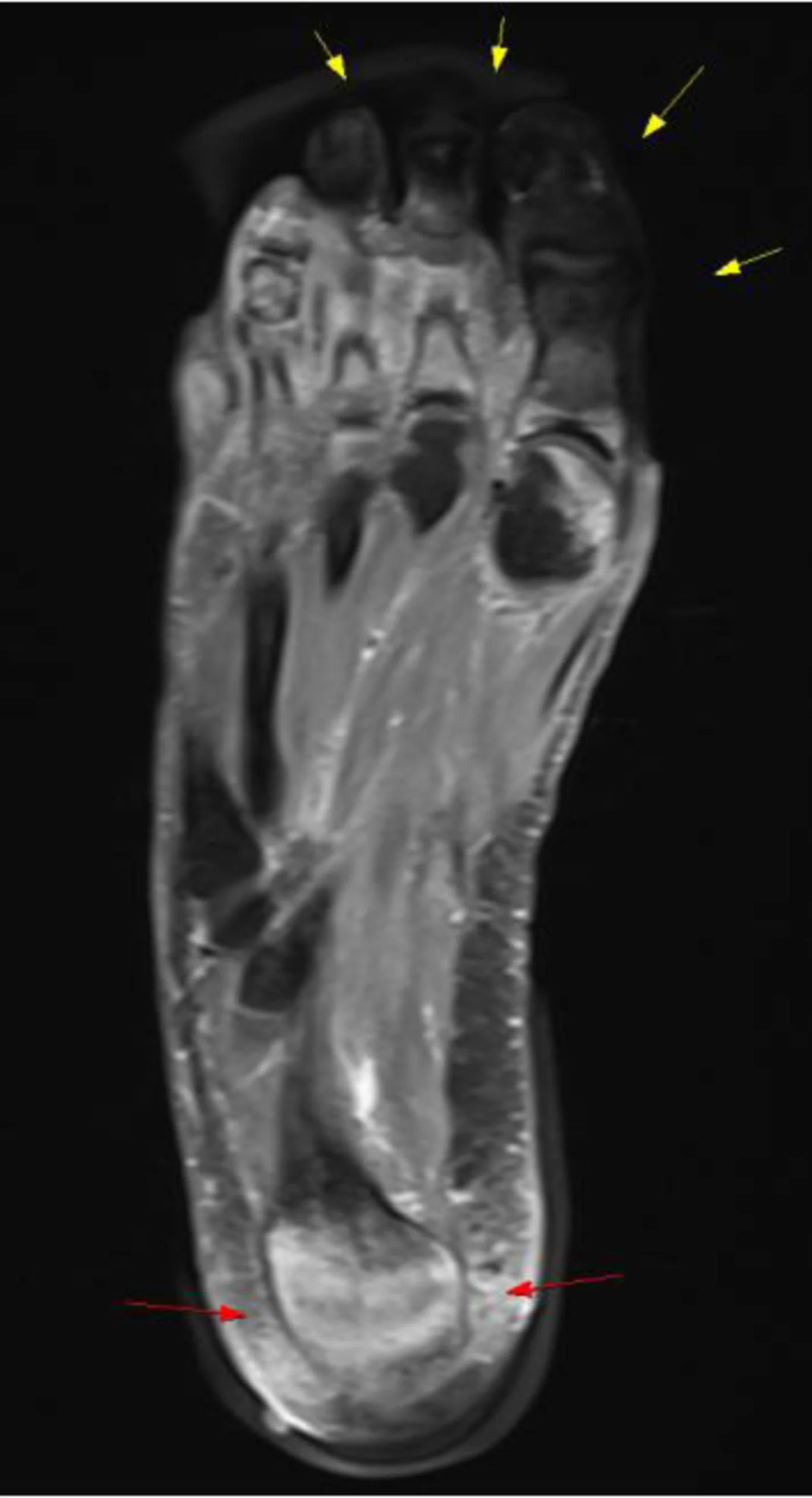
Contrast-enhanced fat-suppressed T1-weighted axial magnetic resonance image of the right foot. The first to third toes show nonenhancing low signal intensity compatible with established bone and soft-tissue necrosis (yellow arrows). The plantar soft tissue and marrow around the calcaneal tuberosity demonstrate bone marrow inflammation and edema (red arrows).

On the left side, MRI and CT showed cold-injury–related bone marrow inflammation in the calcaneal tuberosity, all phalangeal bones of the first to fifth toes, and the heads of the first and fifth metatarsals, accompanied by diffuse subcutaneous edema of the forefoot. Similar to the right side, focal skin necrosis at the left heel with surrounding soft-tissue edema was identified without extension into the calcaneus, plantar fascia, or Achilles tendon. An insufficiency fracture of the posterolateral calcaneus was also present (Fig. 3). These findings were interpreted as being consistent with frostbite-related osteo-medullary injury, with localized areas of established necrosis and more extensive zones of potentially salvageable tissue.

**Figure 3.**
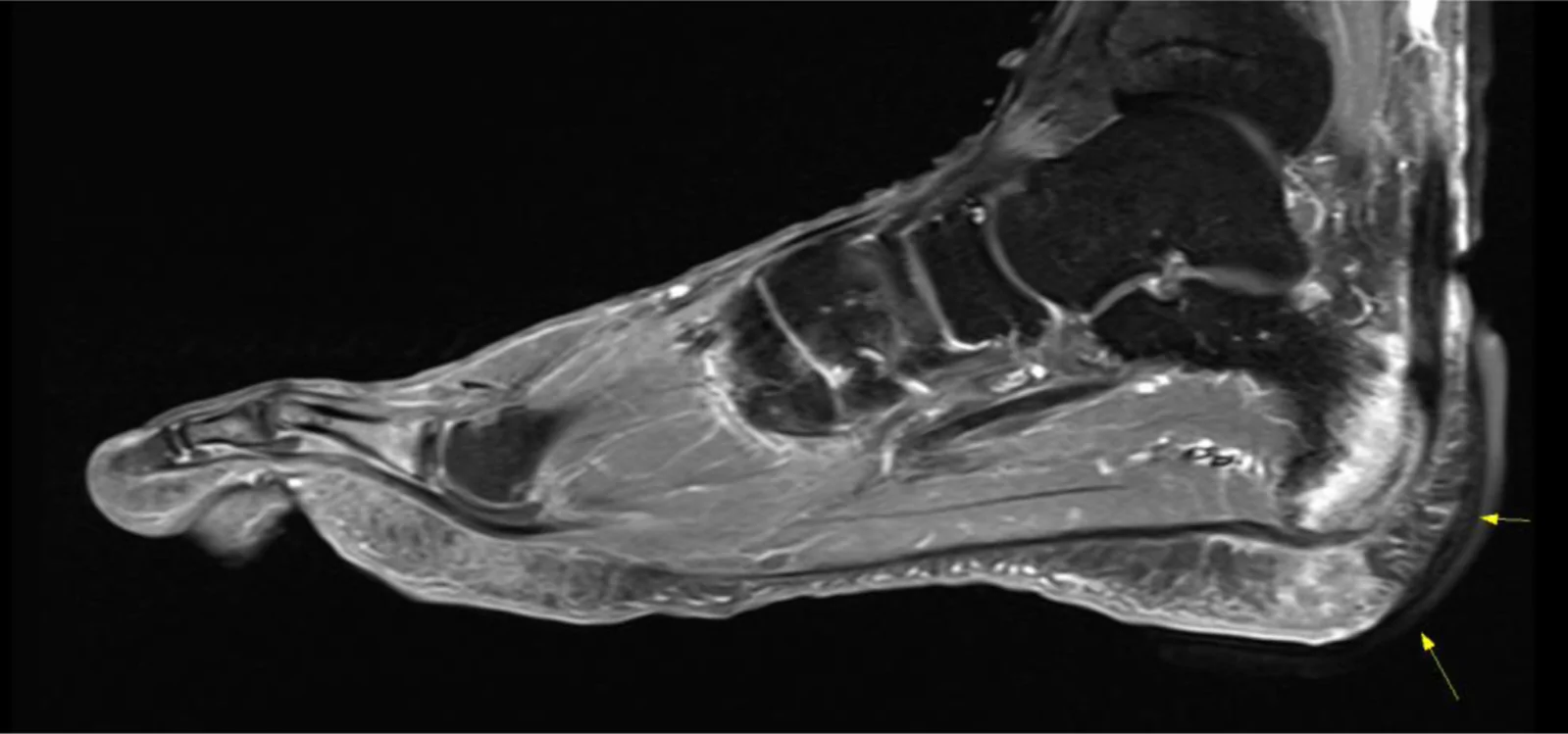
Contrast-enhanced fat-suppressed T1-weighted sagittal magnetic resonance image of the left foot. The posterior heel shows bone marrow inflammation and edema with overlying skin necrosis (yellow arrows).

Given the patient’s young age and preservation of the deep posterior heel structures on imaging, the treating team considered limb salvage a reasonable option. CT angiography demonstrated patent lower-extremity arteries without steno-occlusive lesions and no venous thrombotic occlusion. Primary major amputation, free-flap reconstruction, and RSAF reconstruction were discussed in detail with the patient and his family, including their respective advantages, limitations, postoperative requirements, and the possibility that flap failure could ultimately necessitate amputation. The patient could engage in brief conversations and appeared to understand simple aspects of the situation, but his psychiatric symptoms limited his ability to communicate a clear and consistent treatment preference. The clinical team and family sought to involve him in the discussions and understand his views as far as possible. He did not express opposition to the proposed treatment, although this did not establish a fully informed treatment preference. His family favored attempting limb salvage despite understanding the possibility of subsequent amputation. Considering the bilateral heel defects and uncertainty regarding cooperation with postoperative monitoring and flap protection, together with the additional concern of antipsychotic-associated thrombotic risk, the team selected staged RSAF reconstruction rather than free tissue transfer. The right foot was treated first, with the left-sided reconstruction planned after stabilization. His mother signed the surgical consent form.

The patient underwent amputation of the right first, second, and third toes. The necrotic plantar heel soft tissue on the right side was thoroughly debrided, and the resulting posterior heel defect was reconstructed using a RSAF (Fig. 4). The skin paddle was designed over the posterior calf along the sural neurovascular axis, with its dimensions and pedicle length tailored to the defect and the required arc of rotation. The pivot point was located approximately 5 cm proximal to the tip of the lateral malleolus to preserve the distal peroneal perforators. The flap was elevated from proximal to distal in the subfascial plane, incorporating the sural nerve and small saphenous vein within a broad adipofascial pedicle. The intervening skin was incised to allow transfer of the flap to the heel. The flap was inset without undue tension or pedicle compression, and the skin along the transfer route was subsequently sutured. The donor site was closed primarily where feasible, and the remaining defect was covered with a split-thickness skin graft. After procedure, gauze dressings and a splint were applied, with splinting maintained for approximately 2 to 3 weeks. For the first postoperative week, the patient was maintained exclusively in the prone position, including during meals, to minimize the possibility of inadvertent compression of the flap pedicle. Lateral positioning was not used during this period. Continuous one-to-one observation was provided by nursing staff or a caregiver. Given his earlier attempts to leave the bed and bear weight, wrist restraints were temporarily reapplied for the first 1 to 2 days after surgery as a precaution against unplanned movements that could compromise flap protection during prone positioning. They were removed once he demonstrated sufficient cooperation with positioning instructions. The same brief period of wrist restraint was used after the contralateral reconstruction. In the immediate postoperative period after the first operation, laboratory tests showed only mild, transient elevation of the white blood cell (WBC) count and C-reactive protein (CRP) levels, with CRP values ranging from 20 to 30 mg/L, without other significant abnormalities. Intraoperative tissue cultures from the right-foot wounds yielded methicillin-resistant Staphylococcus aureus (MRSA) and Enterococcus faecalis. Based on these results, intravenous vancomycin was administered for 3 weeks following the right-sided operation, extending to around the time of the left-sided reconstruction, in consultation with the infectious disease service. No systemic signs of sepsis were documented. One week after surgery, the patient was allowed to adopt a supine position with careful padding and strict instructions to avoid direct pressure on the reconstructed heel.

**Figure 4.**
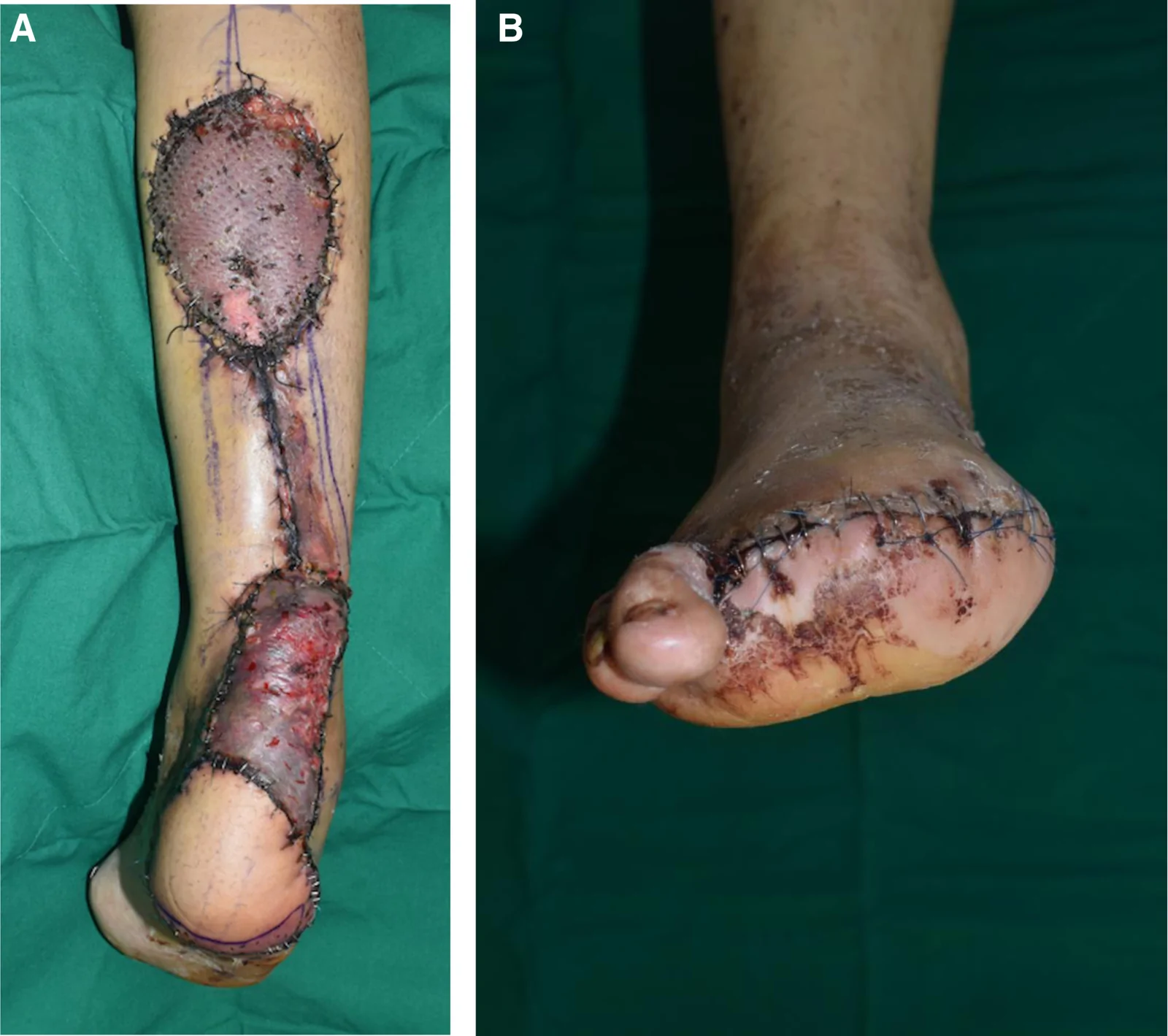
Postoperative views after right heel reconstruction and toe amputation. (A) Posterior view of the right lower leg and heel showing a reverse sural artery flap inset into the posterior heel defect, with the proximal calf donor site covered by a split-thickness skin graft. (B) Anterior view of the right forefoot demonstrating primary closure following amputation of the first to third toes.

The second operation was planned after the first surgical site showed steady healing, stable viability without signs of compromise, and the patient’s cooperation with positioning and non-weight-bearing instructions had improved sufficiently. Three weeks after surgery, the patient underwent debridement of necrotic tissue of the left great toe with split-thickness skin grafting and debridement of the left heel followed by reconstruction with a RSAF (Fig. 5). The same flap elevation and transfer technique was used on the left side, and gauze dressings and a splint were applied. The same positioning protocol was followed after the left-sided reconstruction, with exclusive prone positioning, including during meals, for the first postoperative week. Over the following days, WBC counts remained within the normal range, and CRP values persisted in the 10 mg/L range without other notable laboratory abnormalities. Antibiotic therapy was discontinued after the left-sided reconstruction on the recommendation of the infectious disease service. No new or worsening surgical-site infection was observed during the first 2 weeks after either procedure. One week after surgery, he was again permitted to lie supine with continued precautions to prevent external compression of the flap. After each reconstruction, bed rest was maintained for approximately 1 week, after which wheelchair transfers for toileting were permitted. He was otherwise instructed to remain in bed as much as possible. Standing and walking were not permitted through the last documented outpatient follow-up.

**Figure 5.**
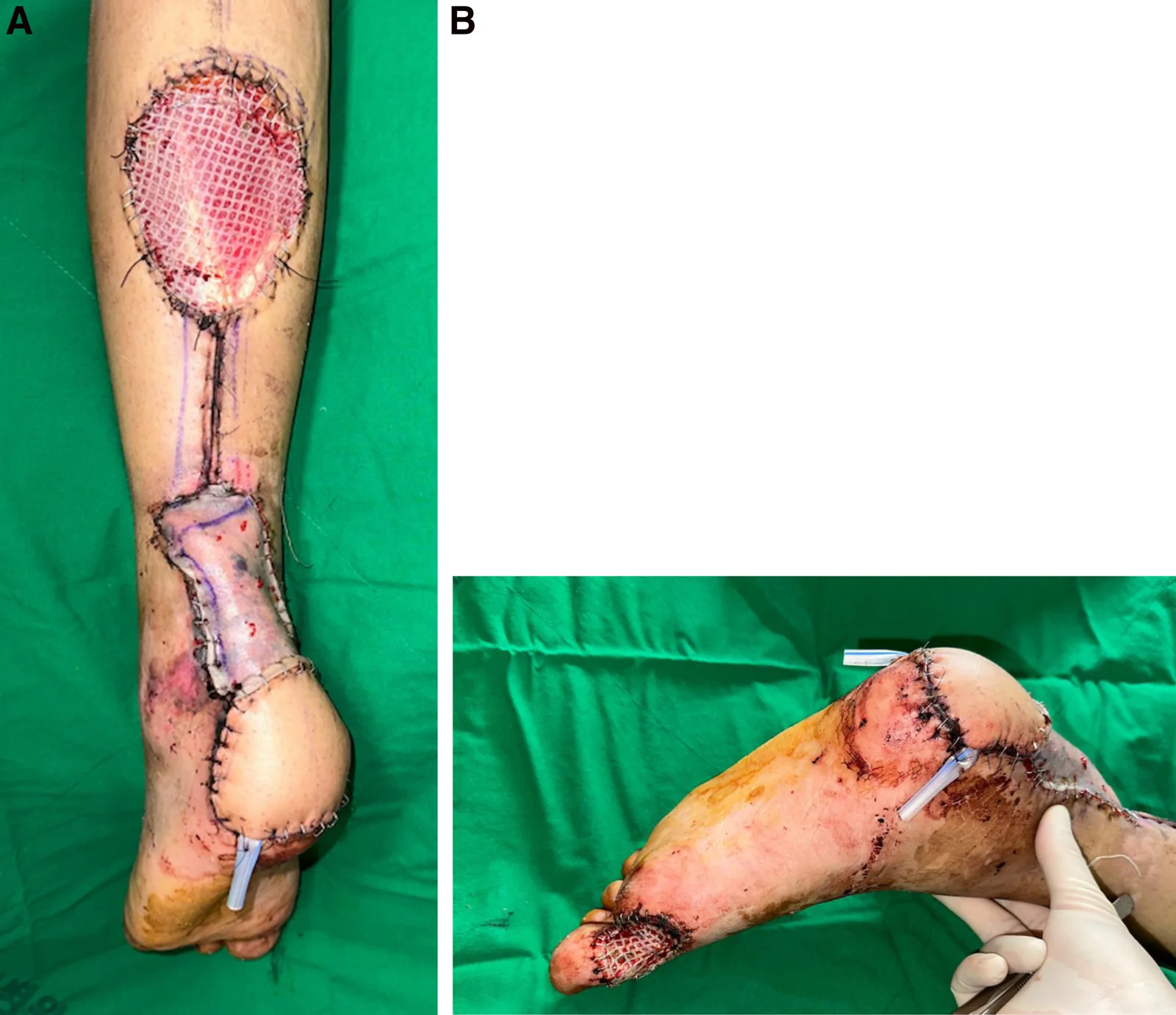
Postoperative views after left heel reconstruction and first toe coverage. (A) Posterior view of the left lower leg showing a reverse sural artery flap inset into the posterior heel defect, with the proximal calf donor site covered by a split-thickness skin graft. (B) Lateral view of the left foot demonstrating split-thickness skin grafting of the first toe.

After each reconstruction, physicians directly assessed the flap every 2 hours during the initial postoperative period. Monitoring included skin color, warmth to palpation, capillary refill following blanching, and Doppler assessment of blood flow. From postoperative day 4, the interval was extended to approximately every 6 hours as the flap was considered clinically stable, and this schedule was maintained through postoperative day 10. Thereafter, assessments were performed during ward rounds and dressing changes, with additional bedside checks as feasible. Both flaps showed mild venous congestion during the first 1 to 2 postoperative days. As the clinical perfusion assessments remained reassuring and Doppler signals were maintained, the flaps were observed without additional intervention, and the congestion subsequently resolved.

Throughout hospitalization, his psychotic symptoms gradually came under control with antipsychotic medication, although his level of insight into his illness remained limited. Three weeks after the left foot surgery, both heel flaps remained clinically viable, and neither flap had required revision or flap-related reoperation; the patient was subsequently discharged. Two weeks after discharge, outpatient follow-up examination showed viable skin paddles in both heel flaps, with no total flap loss, and small residual superficial wounds remained along portions of the flap inset and pedicle incision lines (Fig. 6). At this visit, the patient used a wheelchair for mobility and remained under instructions to avoid standing and weight bearing to protect the reconstructed heels. Standing, weight-bearing capacity, and ambulation were therefore not directly assessed. The patient did not return for further clinical evaluation and could not be contacted; therefore, no longer-term clinical or functional follow-up data were available.

**Figure 6.**
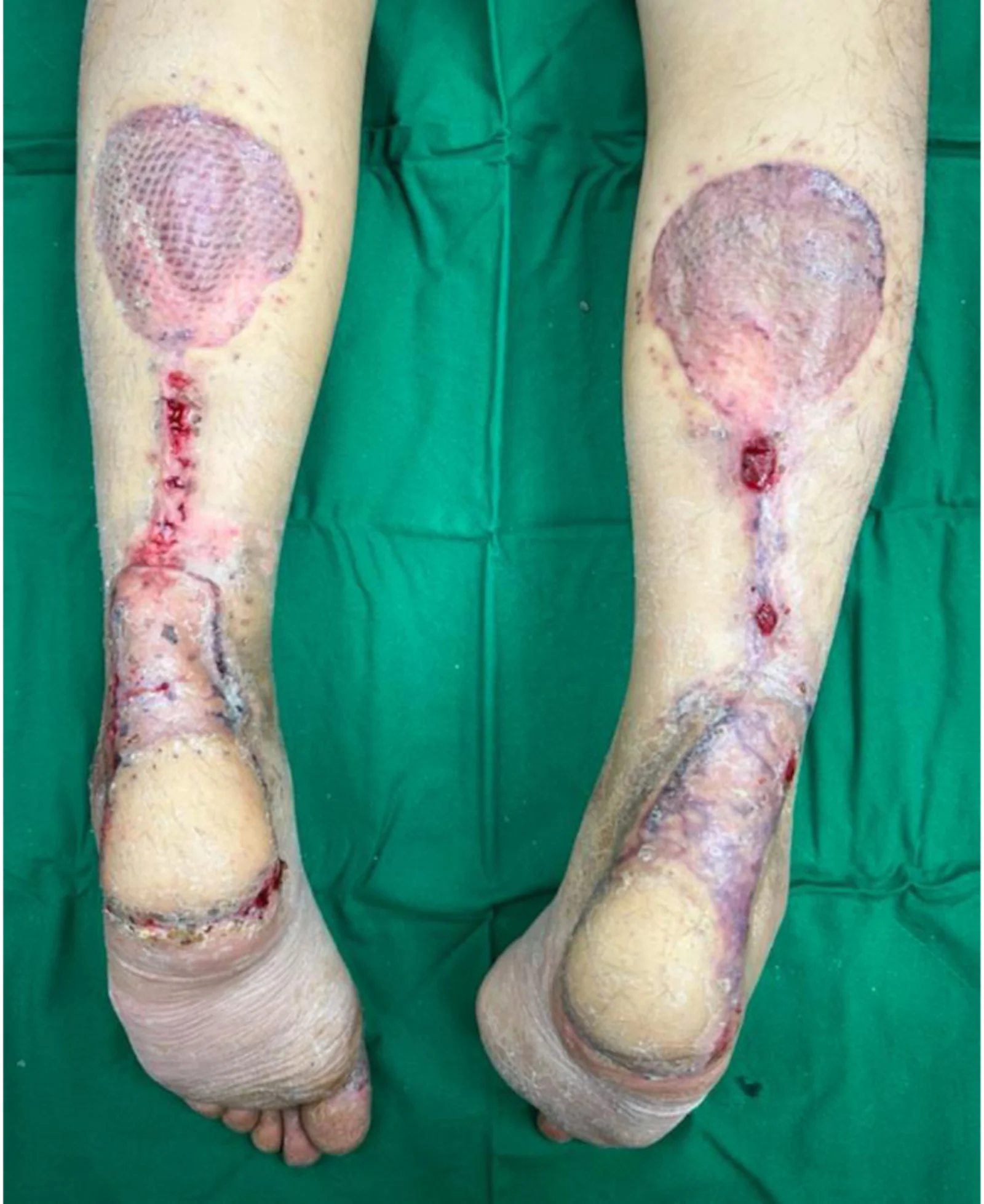
Outpatient follow-up 2 weeks after discharge showing bilateral posterior views of the lower legs and heels. Both heel flap skin paddles remained viable without total flap loss. Small residual superficial wounds were present along portions of the flap inset and pedicle incision lines.

## 3. Discussion

Severe frostbite remains a limb-threatening injury in which the eventual functional outcome is often determined not only by the depth of tissue freezing but also by who is injured and how promptly definitive care can be delivered. In our patient, acute psychosis directly precipitated prolonged barefoot exposure to subzero temperatures, followed by delayed definitive reconstruction and repeated attempts at unprotected weight bearing despite instructions, creating a high-risk setting for deep, bilateral, weight-bearing injury and for postoperative nonadherence. This clinical context is important because the reconstructive “best” option on paper may become the least reliable option in practice when safe immobilization and close monitoring cannot be assured.

Contemporary frostbite management emphasizes rapid rewarming, selective use of thrombolysis within narrow time windows to improve tissue salvage in appropriately selected patients.^[4]^ In our case, the patient presented after a prolonged interval from the exposure, and imaging demonstrated a mixed pattern of established toe necrosis with more localized posterior heel skin necrosis and preservation of deeper heel structures, which supported a limb-salvage rather than primary major amputation approach. This scenario is typical of many real-world frostbite referrals in which early reperfusion options are no longer feasible and reconstruction is required to restore durable plantar coverage.

A central feature of this case is that frostbite occurred in the setting of acute psychosis and was followed by a prolonged reconstructive course while psychiatric symptoms were still being stabilized. Frostbite is not merely an environmental accident; multiple studies have shown that it clusters in socially and psychiatrically vulnerable populations. In particular, Endorf et al highlighted that biopsychosocial factors – including mental illness – are recognized risk factors for sustaining frostbite, and they explored how such factors may also influence complications and readmissions after cold injury.^[5]^ Population-level data similarly identified mental health disorders, homelessness, and drug or alcohol abuse as factors associated with frostbite compared with isolated hand or foot burns.^[6]^ However, the available literature has focused primarily on susceptibility to frostbite, amputation, complications, and readmission rather than flap selection or postoperative adherence after reconstruction. Therefore, evidence directly guiding reconstructive decision-making in psychiatric populations remains limited.

From a reconstructive standpoint, bilateral weight-bearing heel involvement creates a uniquely narrow therapeutic window: the “ideal” reconstructive solution (e.g., microsurgical free tissue transfer for large plantar defects) must be weighed against the real-world requirements of strict positioning, prolonged off-loading, vigilant monitoring, and consistent cooperation. In this patient, limited insight and disorganized behavior translated into repeated attempts at unplanned ambulation and difficulty adhering to instructions – factors that could plausibly increase the probability of mechanical trauma, pressure-related breakdown, or catastrophic flap compromise if a microsurgical strategy were chosen. Consequently, the reconstructive plan prioritized a robust, regional option that could provide durable coverage without microvascular anastomosis and that could be implemented in a staged fashion while psychiatric stabilization progressed. In this context, the RSAF is widely used for distal lower-extremity defects, and a recent multicenter retrospective analysis supports that RSAF remains a reliable and effective option for lower-extremity reconstruction, with clinically meaningful patterns of success and failure that can inform patient selection and perioperative planning.^[7]^ In a heel-specific series of 23 nonsensate RSAFs with a mean follow-up period of 57 months, all patients returned to activities of daily living, 86% were able to use normal footwear, two developed transient superficial ulcers, and no full-thickness ulceration was reported.^[8]^ Taken together, these findings support RSAF as a viable option for heel reconstruction while emphasizing the need for continued surveillance to assess long-term durability and ulceration.

Comparative evidence directly evaluating RSAF against free flaps for weight-bearing heel reconstruction remains sparse. In a small retrospective study of weight-bearing plantar defects, Struckmann et al found no significant difference in functional outcomes between 12 free and 9 pedicled flaps; however, the study was neither restricted to heel defects nor specific to RSAF.^[9]^ Heel-specific series have reported return to activities of daily living and use of normal footwear after RSAF reconstruction and postoperative ambulation after free-flap reconstruction; however, substantial differences in indications and patient comorbidity do not permit conclusions regarding the relative effectiveness of the two approaches.^[8,10]^ Accordingly, our selection of staged RSAFs should not be interpreted as evidence that RSAF is generally superior to free tissue transfer. Rather, in this patient, bilateral weight-bearing defects and unreliable cooperation with postoperative monitoring and off-loading during acute psychosis shifted the risk–benefit balance toward a staged regional approach that avoided dependence on microvascular anastomoses.

Another consideration was systemic thrombotic risk in the setting of antipsychotic therapy. Many patients with schizophrenia require antipsychotics, and meta-analytic evidence has associated antipsychotic exposure with an increased risk of VTE.^[3]^ The patient’s regular antipsychotic regimen included olanzapine, quetiapine, and paliperidone. Although the underlying mechanisms remain incompletely understood, sedation associated with agents such as olanzapine and quetiapine may reduce mobility and contribute to venous stasis.^[11]^ Paliperidone can elevate prolactin through dopamine D_2_ receptor antagonism, and antipsychotic-associated hyperprolactinemia has been linked to increased markers of coagulation activation.^[12]^ These represent proposed mechanisms rather than demonstrated causes of thrombosis. The treating team considered the reported medication-associated VTE risk alongside bilateral heel defects and uncertain adherence to postoperative protection when selecting staged RSAF reconstruction. However, systemic VTE and microvascular anastomotic thrombosis are distinct outcomes, and the cited evidence does not establish that these medications increase free-flap thrombosis or that RSAF reduces systemic VTE risk. Antipsychotic exposure was therefore an additional consideration in individualized planning, rather than an independent contraindication to free tissue transfer.

The strengths of management in this case include: deliberate staging that allowed initial stabilization of the first reconstruction and incremental improvement in ward cooperation before undertaking contralateral surgery; objective vascular assessment (e.g., CT angiography) to confirm macrovascular suitability for flap-based reconstruction; and multidisciplinary coordination with psychiatry and infectious disease services to align psychiatric stabilization, infection control, and flap protection within a realistic inpatient care pathway.

This report has several important limitations. A major limitation is the short duration of documented follow-up. Because the patient did not return after the last outpatient visit and could not be contacted, long-term flap durability under weight-bearing conditions, recurrent ulceration or skin breakdown, sensory recovery, pain, ambulatory function, and the need for additional procedures could not be evaluated. Second, the single-case design precludes conclusions regarding comparative effectiveness and limits the generalizability of the observed early outcome to other patients. Third, acute psychiatric symptoms limited the patient’s ability to communicate a clear and consistent treatment preference. Although efforts were made to involve him in the discussions, his own preferences could not be fully established. The selected treatment reflected the clinical team’s assessment of the feasibility of limb salvage and discussions with his family, limiting extrapolation to patients with different clinical circumstances or treatment preferences. Fourth, this was a single-institution experience involving a multidisciplinary inpatient care pathway, and differences in reconstructive expertise, psychiatric support, and postoperative monitoring resources may limit its applicability to other settings. Fifth, although the patient could provide simple reports regarding the presence or absence of pain, acute psychiatric symptoms, limited communication, and the early postoperative immobilization period made comprehensive assessment using validated patient-reported outcome instruments infeasible. Consequently, the patient’s perspective regarding function, satisfaction, and quality of life could not be adequately incorporated, and the subsequent loss to follow-up prevented later assessment. In addition, adherence risk and postoperative behavior could not be quantified prospectively.

The main lesson from this case is that reconstructive choice should account not only for defect characteristics but also for the feasibility of postoperative protection. In this patient, acute psychosis made reliable cooperation with intensive microvascular monitoring and postoperative protection uncertain; therefore, staged RSAF reconstruction was selected as a regional option without microvascular anastomosis. Flap protection was supported by prone positioning, continuous one-to-one observation by nursing staff or a caregiver, temporary wrist restraints during the first 1 to 2 days after each operation, and heel padding after supine positioning. The more severely injured right foot was treated first, and contralateral reconstruction proceeded only after the first flap was clearly viable and cooperation with positioning and non-weight-bearing instructions had improved.

## 4. Conclusion

This case illustrates that bilateral, limb-threatening foot frostbite in a patient with schizophrenia can be managed successfully in the short term with a staged reconstructive strategy that prioritizes flap reliability and postoperative feasibility. However, longer-term follow-up and further studies are needed to validate the durability and generalizability of this approach.

## Author contributions

**Conceptualization:** Jae Hyun Kim.

**Writing – original draft:** Jae Hyun Kim, Myeong Seop Lee.

**Data curation:** Myeong Seop Lee.

**Supervision:** Chan Min Chung.

**Writing – review & editing:** Chan Min Chung.

## References

[1] Essien SK Chireh B Steinberg C Omondi P Zucker-Levin A. Psychosocial and personal predisposing factors of frostbite injury and associated amputation: a systematic review. Inj Epidemiol. 2024;11:62. 10.1186/s40621-024-00546-w PMC11542192 39511653

[2] Donski PK Fogdestam I. Distally based fasciocutaneous flap from the sural region. a preliminary report. Scand J Plast Reconstr Surg. 1983;17:191–6. 10.3109/02844318309013118 6673085

[3] Di X Chen M Shen S Cui X. Antipsychotic use and risk of venous thromboembolism: a meta-analysis. Psychiatry Res. 2021;296:113691. 10.1016/j.psychres.2020.113691 33421839

[4] Wibbenmeyer L Lacey AM Endorf FW. American Burn Association clinical practice guidelines on the treatment of severe frostbite. J Burn Care Res. 2024;45:541–56. 10.1093/jbcr/irad022 37045447

[5] Endorf FW Alapati D Xiong Y. Biopsychosocial factors associated with complications in patients with frostbite. Medicine (Baltim). 2022;101:e30211. 10.1097/MD.0000000000030211 PMC9410586 36042625

[6] Endorf FW Nygaard RM. Socioeconomic and comorbid factors associated with frostbite injury in the United States. J Burn Care Res. 2022;43:646–51. 10.1093/jbcr/irab162 34432022

[7] Abualhaj S Abualhaj M Dawod MS. Reverse sural artery flap for lower extremity reconstruction: a multicenter retrospective analysis of success and failure patterns. J Orthop Traumatol. 2025;26:53. 10.1186/s10195-025-00860-z PMC12343409 40794315

[8] Park JH Choi IC Hong TC Kang JW Park JW. Reconstruction of the weight-bearing heel with nonsensate reverse sural artery flaps. Injury. 2021;52:1993–8. 10.1016/j.injury.2021.04.007 33867149

[9] Struckmann V Hirche C Struckmann F. Free and pedicled flaps for reconstruction of the weightbearing sole of the foot: a comparative analysis of functional results. J Foot Ankle Surg. 2014;53:727–34. 10.1053/j.jfas.2014.06.009 25179455

[10] Sayyed AA Towfighi P Deldar R Attinger CE Evans KK. Free flap reconstruction of plantar weight-bearing heel defects: long-term functional and patient-reported outcomes. Microsurgery. 2022;42:538–47. 10.1002/micr.30889 35394669

[11] Masopust J Bazantova V Kuca K Klimova B Valis M. Venous thromboembolism as an adverse effect during treatment with olanzapine: a case series. Front Psychiatry. 2019;10:330. 10.3389/fpsyt.2019.00330 PMC6529840 31156478

[12] Ishioka M Yasui-Furukori N Sugawara N Furukori H Kudo S Nakamura K. Hyperprolactinemia during antipsychotics treatment increases the level of coagulation markers. Neuropsychiatr Dis Treat. 2015;11:477–84. 10.2147/NDT.S75176 PMC4348124 25750528

